# Amelioration of nephrotoxicity by targeting ferroptosis: role of *NCOA4*, *IREB2*, and *SLC7a11* signaling

**DOI:** 10.1590/1414-431X2024e13116

**Published:** 2024-10-07

**Authors:** N. Sharawy, B.E. Aboulhoda, M.M. Khalifa, G.N. Morcos, S.A.A.G. Morsy, M.A. Alghamdi, I.M. Khalifa, W.A. Abd Algaleel

**Affiliations:** 1 Department of Physiology, Faculty of Medicine, Cairo University, Cairo, Egypt; 2 Department of Anatomy and Embryology, Faculty of Medicine, Cairo University, Cairo, Egypt; 3 Department of Biochemistry, Faculty of Medicine, Cairo University, Cairo, Egypt; 4 Department of Human Physiology, College of Medicine, King Saud University, Riyadh, Kingdom of Saudi Arabia; 5 Department of Basic Medical Sciences, Faculty of Medicine, King Salman International University, South Sinai, Sinai, Egypt; 6 Pathological Sciences Department, MBBS Program, Fakeeh College for Medical Sciences, Jeddah, 21461, Saudi Arabia; 7 Department of Clinical Pharmacology, Faculty of Medicine, Alexandria University, Alexandria, Egypt; 8 College of Medicine, King Khalid University, Abha, 62529, Saudi Arabia; 9 Genomics and Personalized Medicine Unit, The Center for Medical and Health Research, King Khalid University, Abha, 62529, Saudi Arabia; 10 Clinical Sciences Department, MBBS Program, Fakeeh College for Medical Sciences, Jeddah, 21461, Saudi Arabia; 11 Department of Internal Medicine, Faculty of Medicine, Ain Shams University, Cairo, Egypt; 12 Faculty of Medicine, Galala University, Suez, Egypt

**Keywords:** Ferroptosis, Ferritinophagy, Iron, Kidney, Cisplatin, Deferiprone

## Abstract

Nephrotoxicity is a common complication that limits the clinical utility of cisplatin. Ferroptosis is an iron-dependent necrotic cell death program that is mediated by phospholipid peroxidation. The molecular mechanisms that disrupt iron homeostasis and lead to ferroptosis are yet to be elucidated. In this study, we aimed to investigate the involvement of nuclear receptor coactivator 4 (*NCOA4*), a selective cargo receptor that mediates ferroptosis and autophagic degradation of ferritin in nephrotoxicity. Adult male Sprague-Dawley rats were randomly-assigned to four groups: control group, cisplatin (Cis)-treated group, deferiprone (DEF)-treated group, and Cis+DEF co-treated group. Serum, urine, and kidneys were isolated to perform biochemical, morphometric, and immunohistochemical analysis. Iron accumulation was found to predispose to ferroptotic damage of the renal tubular cells. Treatment with deferiprone highlights the role of ferroptosis in nephrotoxicity. Upregulation of *NCOA4* in parallel with low ferritin level in renal tissue seems to participate in iron-induced ferroptosis. This study indicated that ferroptosis may participate in cisplatin-induced tubular cell death and nephrotoxicity through iron-mediated lipid peroxidation. Iron dyshomeostasis could be attributed to *NCOA4*-mediated ferritin degradation.

## Introduction

Cisplatin accumulates in proximal tubules and induces renal structural and functional impairment. The clinical use of cisplatin (Cis) is often limited by its nephrotoxicity. The pathogenesis of nephrotoxicity is complex and involves numerous pathways, among which oxidative stress is the best known to induce tubular necrosis. However, the molecular mechanisms by which iron overload develops and contributes to cisplatin-induced tubular necrosis are yet to be fully understood.

Iron toxicity plays a crucial role in proximal tubular injury. The ability of iron to gain and lose electrons suits iron as a catalyst for free radical-generating reactions and formation of hydroxyl radicals. Overproduction of hydroxyl radicals does not only compromise lipid and protein content but also contributes to oxidative DNA damage, all of which are involved in cisplatin-induced tubular necrosis (1).

The Nomenclature Committee on Cell Death (NCCD) defined ferroptosis as “a form of regulatory cell death initiated by oxidative perturbations of the intracellular microenvironment that is under the constitutive control of glutathione peroxidase 4 (GPX4) and can be inhibited by iron chelators” (2).

Emerging evidence revealed that ferroptosis is a new form of programmed necrosis that requires an iron-dependent accumulation of reactive oxygen species (ROS) with concomitant depletion of antioxidant defenses, mainly cystine-glutamate antiporter (SLC7A11), which imports cystine, an essential substrate for the synthesis of glutathione. Furthermore, low levels of glutathione peroxidase 4 (GPX4), an enzyme needed to eliminate lipid ROS, could be implicated in iron-induced ferroptosis (3). Participation of iron overload in ferroptosis is also supported by consecutive studies displaying mitigation of ferroptosis-induced cell death by iron chelators (4,5).

Ferritin is a cellular protein that tightly binds to iron and limits the amount of free iron required to sustain the metabolic needs. Ferritinophagy is a newly characterized form of autophagy whereby ferritin is degraded in the lysosomes (6,7). In ferritinophagy, the nuclear receptor coactivator 4 (*NCOA4*), an autophagy cargo receptor, is found to bind and degrade ferritin with subsequent release of iron (8,9).

Deferiprone (DEF) is a chelator of small molecular weight. The ability of DEF to chelate intracellular iron is facilitated by its lipophilicity. This iron chelator not only acts as scavenger for free iron but also neutralizes ROS through donating hydrogen, thus ameliorating oxidative stress (10).

The role of ferroptosis and ferritinophagy in the context of cisplatin-induced nephropathy has not yet been elucidated. Accordingly, in this study, a novel hypothesis was raised that cisplatin promotes iron toxicity via upregulation of *NCOA4*, which would exacerbate iron release from ferritin and promote ferroptosis-induced oxidative stress and pro-inflammatory polarization of macrophages in renal tissue. We also hypothesized that the usage of DEF could attenuate cisplatin-induced nephrotoxicity and enhance macrophage switch into the anti-inflammatory macrophage phenotype.

## Material and Methods

### Animals

The study was performed according to the guidelines of Cairo University Animal Ethical Committee (protocol number: CU/III/F/14/20). Forty adult male Sprague-Dawley rats (weighing 150-200 g) were purchased from the Animal House Kasr El Aini, Faculty of Medicine, Cairo University. Animals were housed in groups of five per cage under standard light conditions with free access to food and water.

### Experimental protocol

Rats were randomly assigned to four groups: control vehicle-treated group, cisplatin (Cis) group, deferiprone (DEF) group, and cisplatin with deferiprone (Cis+DEF) co-treated group. In DEF and Cis+DEF groups, rats were gavaged with 200 mg/kg body weight deferiprone (Apotex Inc., Canada) for 10 days and injected with saline or cisplatin on the 4th day (11). Cisplatin was supplied as a 1-mg/mL stock solution (Mylan S.A.S, France) that was diluted in saline and administered intraperitoneally (2 mg/kg *ip*) once daily for 3 days beginning on day 4 of the study. All rats were housed in metabolic cages for 24 h before euthanasia. On the 11th day of the study, animals were anesthetized with a ketamine and xylazine mixture (65 and 7 mg/kg, respectively, *ip*) and euthanized. Renal, blood, and urine samples were collected for further biochemical and immunohistochemical analysis.

### Analysis of renal function

The volume of urine was recorded. Creatinine and BUN levels were assayed using colorimetric assay kit according to the recommendations of the manufacturer (Egyptian Company for Biotechnology). Creatinine clearance (CrCl) was calculated as previously reported (12); Ccr (mL/min per kg) = [urinary creatinine (mg/dL) × urinary volume (mL) / serum creatinine (mg/dL)] × [1000 / bodyweight (g)] × [1/1440 (min)]

### Analysis of iron parameters and oxidative stress in renal tissue

Malondialdehyde (MDA) is the most prevalent biomarker for lipid peroxidation (13), and it was quantified using the enzyme-linked immunosorbent assay (Stat Fax 2200, Awareness Technologies, USA). The formation of MDA was measured at a wavelength of 490 to 630 nm. The total glutathione (GSH) levels in renal tissue were measured using assay kits from Awareness Technologies. Glutathione was evaluated based on the reduction of 5,5' dithiobis (2 - nitrobenzoic acid) (DTNB) with GSH to produce a yellow compound, which was measured at 405 nm. The changes in the absorbance were detected at 560 nm. All measurements were performed colorimetrically.

Iron status was assessed using iron, total iron binding capacity (TIBC), and transferrin saturation. Serum and renal iron as well as TIBC were quantified using ELISA assay kits (Mybiosource, USA) at 450nm absorbance by a microtiter plate reader (Thermo Scientific, USA). Transferrin saturation refers to serum iron/TIBC × 100.

### Analysis of serum transferrin and renal ferritin

Concentrations of serum and renal ferritin were determined using available ELISA assays (Abcam^®^, USA) in accordance to the manufacturer's instructions. The transferrin and ferritin present in the samples react with specific antibodies. Unbound proteins were removed by washing, and antibodies conjugated with horseradish peroxidase (HRP) were added. A chromogenic substrate, 3,3',5,5'-tetramethylbenzidine (TMB), was added to assay the enzyme bound to the immunosorbent. The absorbance at 450 nm was measured using the microplate reader (Thermo Scientific). The standard curve was constructed and the concentrations of ferritin and transferrin were estimated.

### Real-time quantitative polymerase chain reaction (RT-qPCR)

*NCOA4*, *IREB2*, *SLC7A11*, *GPX4*, and *FTH1* gene levels were quantified via RT-qPCR assay. Total RNAs were extracted from renal tissue using Qiagen QIAamp (USA). The QuantiTect Reverse Transcription kit (USA) was used for cDNA synthesis and the QuantiFast SYBR Green PCR kit was used for PCR assay (Qiagen). The two-step PCR cycling protocol was as follows: a denaturation step at 95°C and a combined annealing/extension step at 60°C. Glyceraldehyde-3-phosphate dehydrogenase (GAPDH) was used as a house-keeping gene. For calculation of the relative gene level, the 2^−ΔΔCT^ method was used. The primer sequences are shown in Table 1.

**Table 1:** Sequences of primers used in real-time polymerase chain reaction.

Gene	Primer sequences (5′-3′)
*NCOA4*	
Forward	5′-GGGGAATTCACCGCCATGAATACCTTCCAAGAC-3′
Reverse	5′-GGGGAATTCTCACATCTGTAGAGGAGTTCG-3′
*IREB2*	
Forward	5′- GTGACACTGTCTCTGTTCGT-3′
Reverse	5′- TGTGTAACCATCCCACTGCC-3′
*SLC7A11*	
Forward	5′- TTCATCCCGGCACTATTTTC-3′
Reverse	5′- CGTCTGAACCACTTGGGTTT-3′
*GPX4*	
Forward	5′- AGGATGTGGATCGTAACGCGTG-3′
Reverse	5′- TCAGAGACATCACAGGTATTTAGG-3′
*FTH1*	
Forward	5′- AAGATGGGTGCCCCTGAAG-3′
Reverse	5-′ CCAGGGTGTGCTTGTCAAAGA -3′
*GAPDH*	
Forward	5′- AGGTCGGTGTGAACGGATTTG-3′
Reverse	5-′ TGTAGACCATGTAGTTGAGGTCA-3′

### Histology and immunostaining analysis

The kidney specimens were carefully dissected, fixed in 10% formol saline solution, and paraffin embedded. Sections of 5-μm thickness were prepared via routine histological preparation. The histopathological lesions including vascular dilatation and congestion, glomerular necrosis and/or sclerosis, tubular vacuolation, intra-luminal hyaline casts, and interstitial inflammatory cellular infiltration were evaluated in hematoxylin and eosin (H&E)-stained sections under 400× magnification. Slides were graded as previously described (14), where score 0 indicates no significant alterations, (+) denotes minimal changes, (++) moderate changes, and score (+++) indicates severe pathological lesions (Table 2).

**Table 2:** Scoring of the severity of histopathological lesions in renal tissue.

	Control	DEF	Cis	Cis+DEF
Dilatation and congestion of the interstitial blood capillaries	-	-	++	+
Glomerular necrosis and/or glomerular capillary tuft retraction with widening of the urinary space	-	-	+++	-
Vacuolation of the tubular epithelial lining and/or intratubular hyaline casts	-	-	++	-
Tubular degeneration and/or inflammatory cellular infiltration	-	-	++	+

DEF: Deferiprone; Cis: cisplatin; -: no changes; +: mild changes; ++: moderate changes; +++: severe changes.

Kidney sections were processed for immunostaining using the standard labelled streptavidin-biotin immunoenzymatic antigen detection procedure according to the manufacturer's instructions via an automated staining protocol (15) using the anti CD163 antibody (EPR19518, ab182422 diluted at 1:100, IHC-P, Abcam^®^) for detection of M2 anti-inflammatory macrophage phenotype. M1 pro-inflammatory macrophages were detected through rabbit monoclonal anti-CD68 antibody (1:50, C68/2160R, ab234401, Abcam^®^). Macrophages positive for CD163 and CD68 appeared as brown deposits. Positive controls were obtained via immunostained lung tissues. Negative controls were obtained through omission of incubation with the primary antibody. CD163 and CD68 immunohistochemically-stained cells were counted morphometrically using the Leica Qwin 500 Image Analyzer Computer System (England) under 400× magnification in 6 microscopic fields inside a standard measuring frame of 85,550 mm.

### Statistical analysis

For comparisons between different groups, ANOVA was performed with Turkey's *post hoc* tests using GraphPad Software 5.0 (USA). Pearson's correlation test was also used to analyze the relationship between iron level and *NCOA4*. Normalized data are reported as means±SE. Significance was defined as P<0.05.

## Results

### Deferiprone reduced renal iron accumulation and ameliorated cisplatin-induced nephrotoxicity

After cisplatin treatment, iron concentration was significantly elevated in renal tissue (Cis *vs* Control, P<0.05). Serum iron, TIBC, transferrin, and transferrin saturation were not significantly changed after cisplatin administration (Cis *vs* Control, P>0.05). However, treatment with DEF significantly reduced serum and renal iron levels but not transferrin levels (DEF *vs* Control and DEF *vs* Cis+DEF, P<0.05) (Table 3).

**Table 3:** Levels of iron and renal function markers in cisplatin-induced nephrotoxicity and the effect of deferiprone (DEF).

	Control	DEF	Cis	Cis+DEF
Creatinine (mg/dL)	0.39±0.06	0.53±0.14^$^	1.81±0.45^$^*	0.59±0.19^#^
BUN (mg/dL)	28.75±0.95	29.50±2.08	75.67±8.80^$^*	57.67±9.66^$*#^
Cr clearance (mL/min/kg)	0.02±0.007	0.01±0.005	0.0002±0.000^$^*	0.01±0.007^$#^
Kidney weight index	0.003±0.0007	0.004±0.0007	0.004±0.002	0.003±0.0008
Plasma transferrin (mg/mL)	4.7±0.6	5.2±0.3	4.9±0.6	5.1±0.3
Serum Iron (μmol/L)	7.5±0.53	7.1±0.92	7.7±0.83	6.55±1.04
TIBC (μmol/L)	129.5±49.7	138.8±52.8	153.8±51.4	161.4±58.9
Transferrin saturation (%)	72.75±10.3	68.00±6.2	70.67±4.3	67.33±4.8
Renal iron (μg Fe/mg tissue weight)	0.84±0.17	0.68±0.42	2.63±0.77^$^*	0.98±0.11^#^

Data are reported as means±SE. ^$^P<0.05 *vs* control, *P<0.05 *vs* DEF, ^#^P<0.05 *vs* Cis (ANOVA). DEF: deferiprone; Cis: cisplatin; BUN: blood urea nitrogen; Cr clearance: creatinine clearance; TIBC: total iron binding capacity.

A significant impairment of renal function with iron overload was observed. This was manifested as depletion of creatinine clearance (Cis *vs* Control, P<0.05) along with a significant increase in creatinine (Cis *vs* Control, P<0.05), and BUN (Cis *vs* Control, P<0.05). These cisplatin-induced changes were significantly reversed by iron scavenging using DEF (Cis+DEF *vs* Cis, P<0.05) (Table 3).

**Figure 1:**
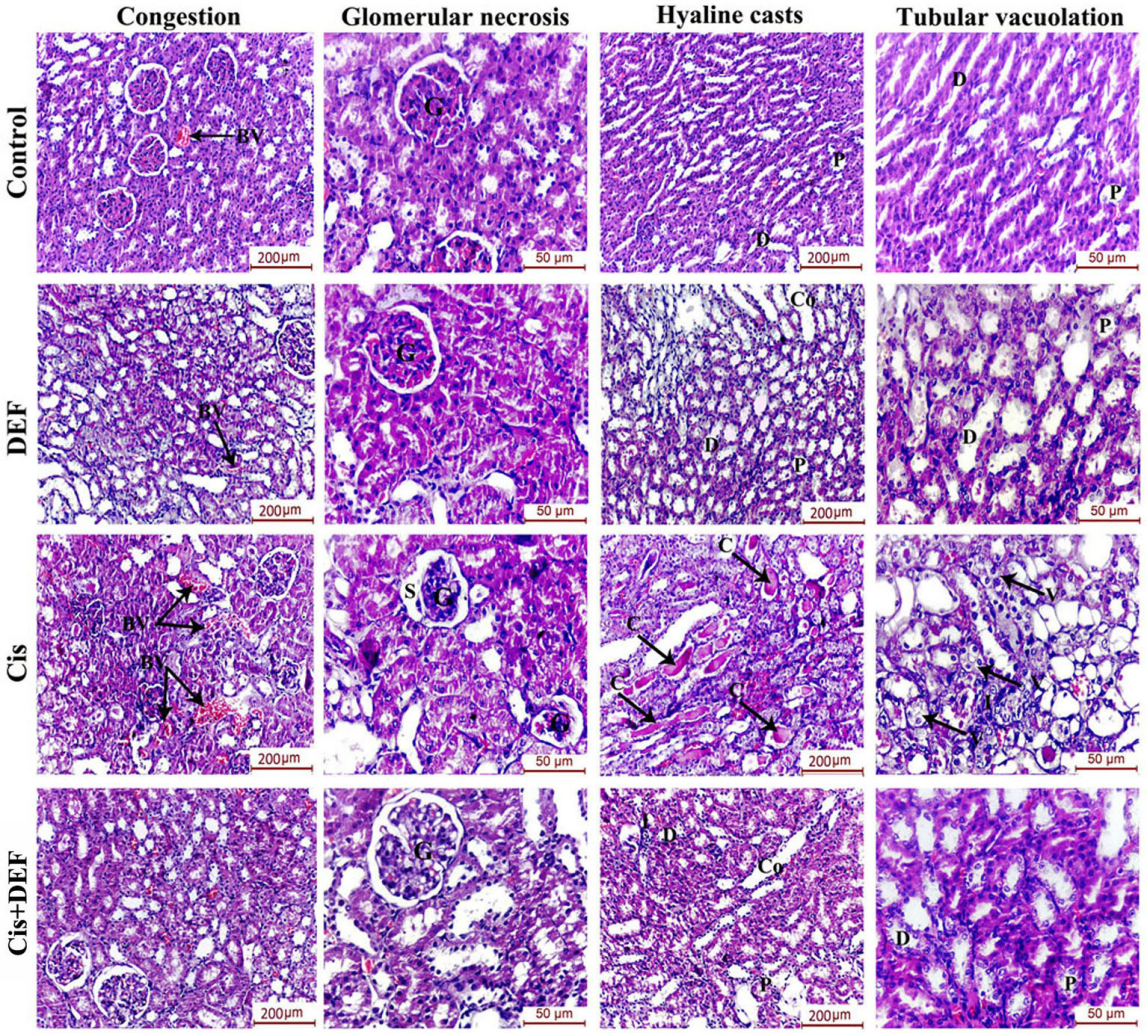
Deferiprone (DEF) mitigated necrosis and improved renal structure after cisplatin (Cis) treatment. H&E-stained sections of the four study groups showing marked pleopathological changes in the kidney of the cisplatin-treated group where obvious dilatation and congestion of the interstitial blood capillaries was observed. Glomeruli appeared degenerated with marked atrophy of the glomerular capillary tuft, widening of the Bowman's space, and irregularity of the Bowman's capsule. The tubular lumina are seen filled with hyaline casts (C) and their epithelial lining appear highly-vacuolated. Some tubules appear degenerated with surrounding inflammatory cellular infiltration (I). Kidney sections of the DEF-treated group demonstrated improvement in renal architecture. The glomerular capillaries appeared healthy with normal urinary space and Bowman's capsule. The proximal (P), distal (D), and collecting (Co) tubules showed improvement of their histological structure with intact epithelial lining and minimal inflammatory infiltration (I). BV: Blood vessels; G: Glomeruli; S: Bowman's space; V: Vacuolation. Scale bars 200 and 50 μm.

To assess the role of DEF in protecting the kidney from cisplatin-induced renal damage, we also studied the renal immune-histopathological changes. While the normal renal tissue revealed normal glomerular and tubular structure, the rats treated with Cis showed features of glomerular degeneration, tubular vacuolization, and interstitial inflammatory cellular infiltration. Pre-treatment with DEF significantly ameliorated the Cis-induced glomerular and tubular necrosis, where the glomeruli exhibited intact capillary tuft and normal urinary space and Bowman's capsule. Tubule architecture was improved, with intact epithelial lining and minimal inflammatory infiltration Figure 1 and Table 2.

### Cisplatin exacerbated ferroptotic necrosis in renal tissue

**Figure 2:**
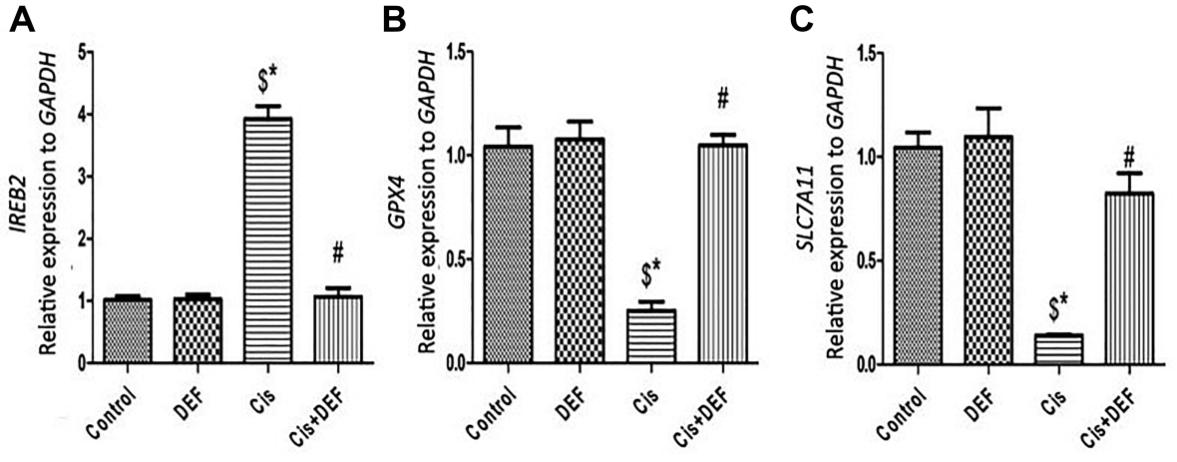
Ferroptosis participated in cisplatin (Cis)-induced nephrotoxicity. **A**-**C**, RT-PCR analysis of expression of ferroptosis-related genes. Cisplatin induced iron-responsive element-binding protein 2 (*IREB2*) (**A**) gene upregulation in parallel with *GPX4* (**B**) and *SLC7A11* (**C**) gene downregulation that was reversed by deferiprone (DEF) co-treatment. Data are reported as means±SE. ^$^P<0.05 *vs* Control, *P<0.05 *vs* deferiprone (DEF), ^#^P<0.05 *vs* cisplatin (Cis) (ANOVA).

**Figure 3:**
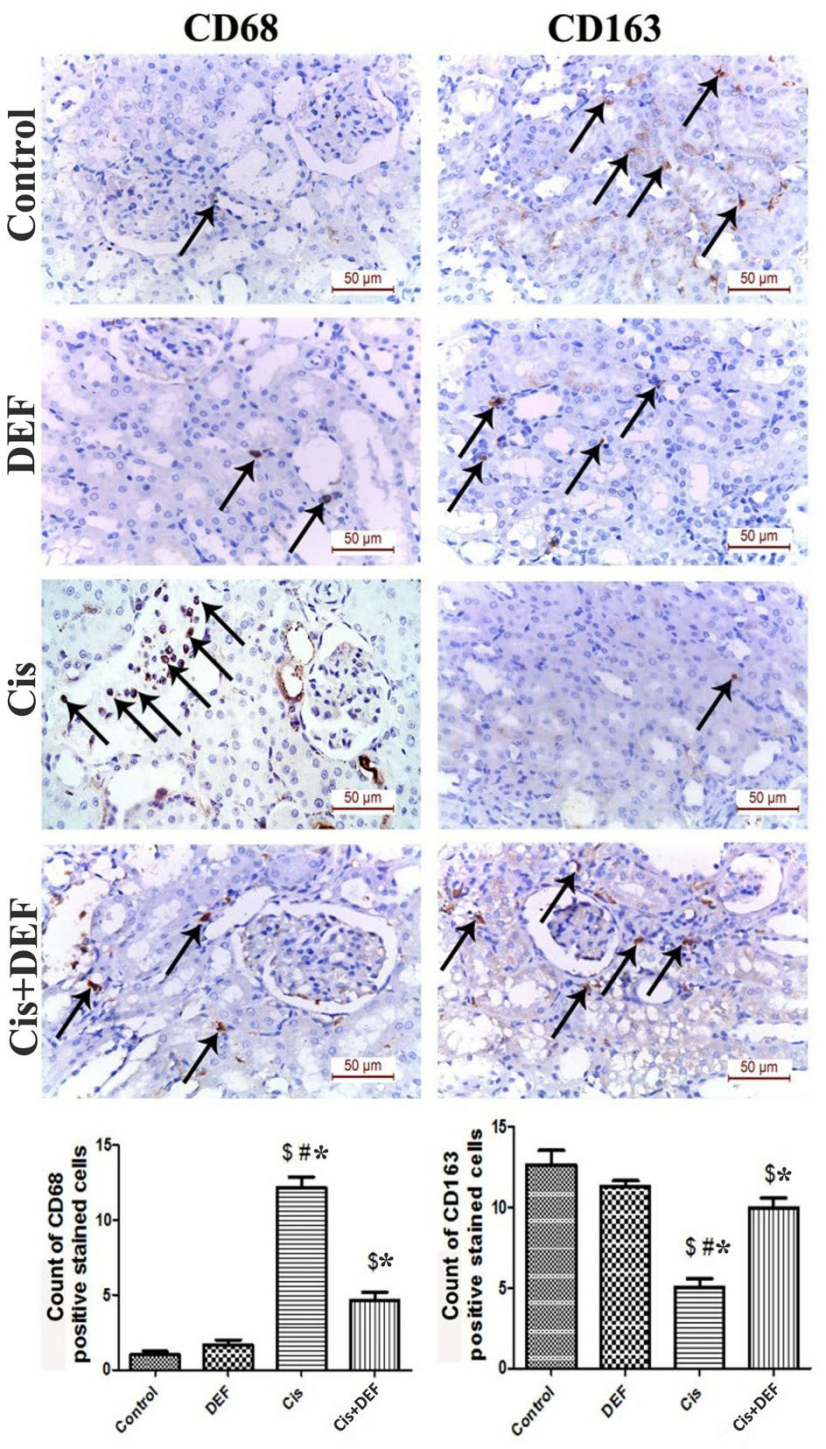
Deferiprone (DEF) switched the macrophage polarization towards the anti-inflammatory phenotype. Representative images of immunohistochemical expression (magnification ×400, scale bar 50 µm) and quantification of the CD68- and CD163-positive macrophage count (arrows) in renal tissue of the cisplatin (Cis) rat model compared to control and DEF-treated group showing significantly increased CD68-positive pro-inflammatory macrophages in the Cis group mainly observed in the interstitial and peri-glomerular areas. CD163-positive anti-inflammatory macrophage count was significantly decreased in the Cis group. Data are reported as means±SE (n=6). ^$^P<0.05 *vs* Control, *P<0.05 *vs* DEF, ^#^P<0.05 *vs* Cis + DEF (ANOVA).

To define the role of cisplatin in controlling ferroptosis, we measured the expression of important genes that regulate iron metabolism and lipid peroxidation, namely *IREB2* (iron-responsive element binding protein 2), *FTH1* (ferritin, heavy polypeptide 1), *GPX4* (glutathione peroxidase 4), and *SLC7A11* (cystine/glutamate transporter). In comparison to the control group, the expression of *IREB2* was significantly upregulated (Cis *vs* Control, P<0.05), while *GPX4* and *SLC7A11* genes were significantly downregulated (Cis *vs* Control, P<0.05) by cisplatin. These alterations were significantly reversed with DEF treatment (Cis+DEF *vs* Cis, P<0.05) (Figure 2). Furthermore, ferroptosis was associated with a switch of macrophages to a pro-inflammatory phenotype, as indicated by significant increases of CD68 and a significant decrease of CD163. Thus, accumulated iron in renal tissue promoted the pro-inflammatory macrophage polarization and further aggravated cisplatin-induced necrosis Figure 3.

### Deferiprone ameliorated cisplatin-induced oxidative stress and lipid peroxidation in renal tissue

**Figure 4:**
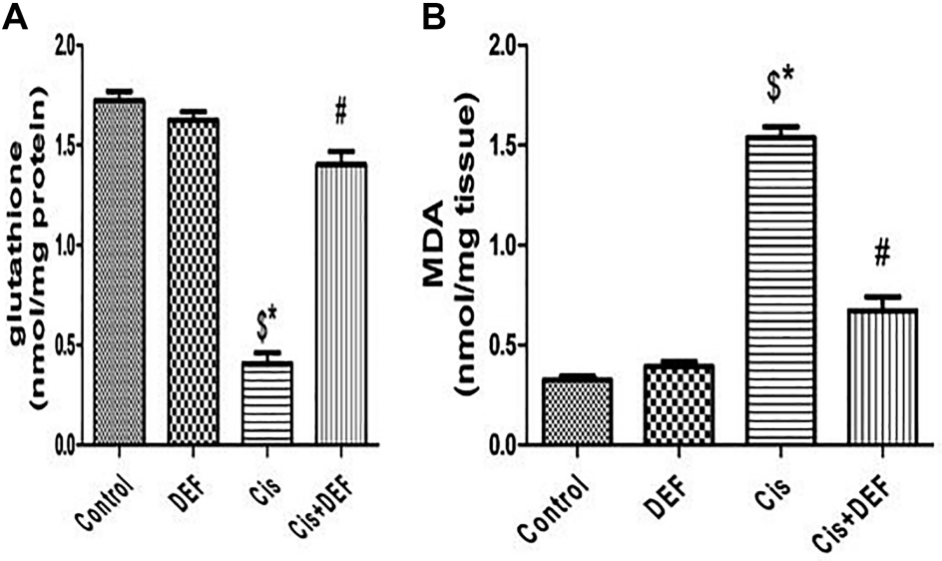
Deferiprone (DEF) mitigated oxidative stress and lipid peroxidation derived by cisplatin (Cis)-induced ferroptosis. **A** and **B**, Quantitative analysis of glutathione and malondialdehyde (MDA). Data are reported as means±SE. ^$^P<0.05 *vs* Control, *P<0.05 *vs* DEF, ^#^P<0.05 *vs* Cis (ANOVA).

To define the molecular mechanisms of action of cisplatin in nephrotoxicity, we measured MDA and GSH as important signaling markers of ferroptosis-induced cell damage. Cisplatin induced a significant depletion of GSH (Cis *vs* Control, P<0.05) in parallel with a significant increase in MDA (Cis *vs* Control, P<0.05). Chelation of iron by DEF significantly improved the negative effects of ferroptosis by a significant upregulation of GSH (Cis+DEF *vs* Cis, P<0.05) and a significant reduction of MDA (Cis+DEF *vs* Cis, P<0.05) (Figure 4).

### Cisplatin exacerbated ferritinophagy and decreased ferritin in renal tissue

**Figure 5:**
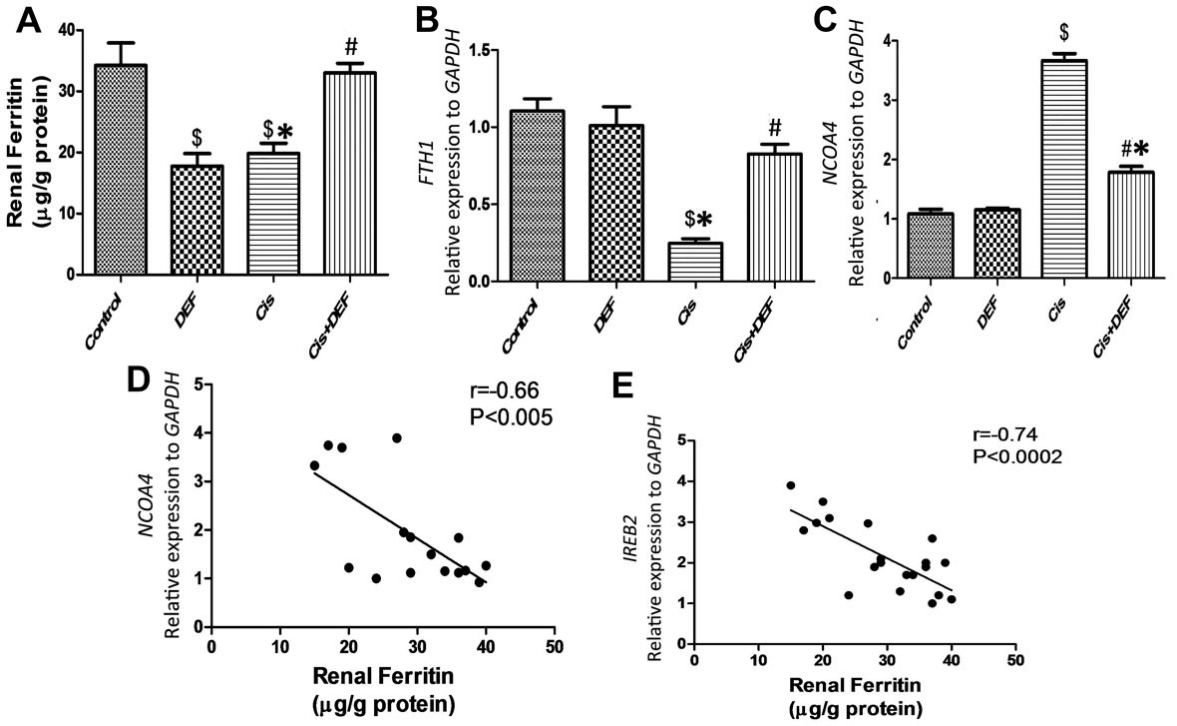
Deferiprone (DEF) attenuated cisplatin (Cis)-induced ferritinophagy. **A**-**C**, Quantitative analysis of ferritin, iron storage protein ferritin heavy chain 1 (*FTH1*), and nuclear receptor coactivator 4 (*NCOA4*) in the renal tissue. **D** and **E**, Pearson correlation analysis between ferritin level in the kidney and NCOA4 (**D**) and iron-responsive element-binding protein 2 (*IREB2*) (**E**) expressions. Data are reported as means±SE. ^$^P<0.05 *vs* Control, *P<0.05 *vs* DEF, ^#^P<0.05 *vs* Cis (ANOVA).

*NCOA4* plays a central role in ferritinophay as well as the main iron storage protein ferritin heavy chain 1 (*FTH1*) that was found to inhibit ferritinophagy and downregulate *NCOA4*. The present results indicated that the expression of *NCOA4* was significantly increased by cisplatin (P<0.05). On the other hand, the expression of *FTH1* was significantly decreased in the cisplatin group. DEF significantly mitigated the expression of *NCOA4* and concomitantly increased *FTH1* and ferritin (Cis+DEF *vs* Cis, P<0.05). Interestingly, in the DEF-treated rat group, scavenging of iron significantly decreased ferritin level and *FTH1* expression while the expression level of *NCOA4* increased significantly (DEF *vs* Control, P<0.05), suggesting that redox status and iron level may influence *NCOA4* expression (Figure 5A-C). Furthermore, ferritin levels were inversely related to NCOA4 and *IREB2* expressions. Taken together, the results suggested that the redox status can influence the expressions of *NCOA4* and *IREB2* in parallel, thus creating an imbalance favoring ferritin breakdown over synthesis (Figure 5D and E).

## Discussion

In the present study, we found that ferritinophagy promoted iron overload in the renal tissues. Iron accumulation aggravates ferroptotic cell death and pro-inflammatory macrophage polarization.

Cisplatin is frequently used to treat various types of malignancies; however, its use is associated with nephrotoxicity. Patients receiving cisplatin chemotherapy frequently develop acute kidney injury (AKI) due to the interaction of cisplatin-induced ROS with different cellular components, causing functional damage to proteins, lipids, and different cellular organelles.

Iron overload is involved in the pathogenesis of various renal diseases, but iron homeostasis in the context of cisplatin-induced nephrotoxicity has not been clearly reported (6,16). Here, for the first time, we demonstrated that ferritophagy is involved in the process of cisplatin-induced nephrotoxicity.

Transferrin-bound iron is the primary source of iron in renal epithelial cells (17). Intracellular iron levels are stabilized by binding with cytosolic ferritin, which functions as an iron nanocage that buffers excess iron to prevent oxidative stress. Ferritin in proximal tubular cells is highly inhibited by upregulation of iron-responsive element (*IREB2*) (18,19). Our data showed that nephrotoxicity was associated with ferritin downregulation and *IREB2* upregulation. Similar to our results, previous studies have shown that low ferritin levels play a role in neurodegenerative disorders and tumor progression (20). The importance of ferritin has also been emphasized by the deletion of the ferritin gene, which led to increased mortality after rhabdomyolysis (21). In the hemorrhagic striata of *IREB2*-knockout mice, ferritin was increased to enhance neuronal protection (22). *IREB2* is also an essential gene for ferroptosis. Oxidative stress may drain the cellular iron pool that stimulates *IREB2* binding to the iron responsive element (*IRE*), thereby regulating the transcription of iron-regulating proteins, including ferritin (23,24).

Ferritin degradation can be modulated by iron levels and redox status via the NCOA4 pathway. Stored iron is released from ferritin by NCOA4 stimulation. This subsequently promotes lysosome-mediated degradation of ferritin (25). *NCOA4* gene ablation in human leukemic K562 cells was found to reduce intracellular free iron as a result of failure of ferritin degradation (26). This is supported by the paradoxical relationship between *NCOA4* and ferritin levels observed in the nephrotoxicity model. Increased *NCOA4* level has also been reported to reduce ferritin levels and promote the migratory potential of malignant ovarian cells (27). Furthermore, *NCOA4* depletion enhanced ferritin accumulation and protected human bronchial epithelium cells from cigarette smoke-induced iron toxicity (28).

Free iron accumulation enhances oxidative stress via the Fenton reaction. Iron can be toxic when it reacts with hydrogen peroxide, thereby initiating oxidative stress chain reactions (29). Ferroptosis is an iron-dependent oxidative stress process that causes necrotic cell death. Our results are consistent with the recent findings reporting that ferritinophagy is closely associated with ferroptosis (9). Ferroptosis is structurally and molecularly distinct from apoptosis and autophagy. Structurally, cells are characterized by intact nuclei, cell membranes, and ruptured mitochondria. The defined biochemical features of ferroptosis include increased levels of lipid peroxidation, tissue iron concentration, and *IREB2* and decreased GPX4 and cystine/glutamate antiporter (SLC7A11), resulting in a synchronization of iron dyshomeostasis and disturbed redox status (30).

We found that impaired antioxidant defense was concomitantly associated with iron-dependent peroxidation, which was alleviated by the administration of DEF. GPX4, SLC7A11, and glutathione significantly participated in ROS clearance. Conditional knockout of GPX4 or SLC7A11 demonstrated a link between ferroptosis, cancer, and neurodegenerative diseases (31,32), and iron and MDA levels were increased in *NCOA4*-overexpressing fibroblast cells (33).

DEF induces better cell permeability; therefore, it significantly lowers the renal iron concentration. Our findings are in a line with Sripetchwandee et al. (34) who reported that DEF could mitigate iron-overload induced neurotoxicity. Similarly, DEF reduced iron accumulation and reversed renal damage in thalassemic mice (35). Contrary to our data, Bloomer et al. (16) found that treatment with an iron chelator prevented oxidative damage but did not attenuate the increased renal iron associated with aging. In our study, DEF replenished the levels of GSH and other endogenous antioxidants. Furthermore, reducing cellular iron inhibits ferroptosis and protects renal tissues from cisplatin-induced peroxidation. Our data supported the previous findings suggesting that iron chelation and scavenging of reactive species by iron chelators are involved in prevention of oxidative damage (35).

In our study, we observed that treatment with DEF did not adversely affect peripheral iron status. Unlike other iron chelators, DEF redistributes excess toxic tissue iron to the bone marrow via transferrin, and the small amounts of excreted iron during treatment could be replaced by dietary iron. Thus, iron deficiency is prevented in patients with Parkinson's and Alzheimer's diseases (36).

The cisplatin-induced nephrotoxicity was prominent in the current study, as shown by impairment of renal function, glomerular degeneration, tubular vacuolization, oxidative stress, glutathione depletion, and MDA increase, in addition to iron accumulation with subsequent ferroptotic damage of the renal tubular cells. One of the known pathophysiologic pathways mediating this tissue damage is the upregulation of *NCOA4* and suppression of *FTH1*. Similar results were described in earlier studies where the treatment with cisplatin induced elevation of ferritin and transferrin receptor-1 expression in addition to renal iron content, oxidative free radicals, and inflammatory mediators' expression, which resulted in renal tissue injury and dysfunction (37,38).

DEF, the iron chelator, suppressed the cisplatin-induced ferroptosis and nephrotoxicity. This was demonstrated by the improvement in the glomerular and tubular architecture, the suppression of the inflammatory infiltrate, and the reduction in the oxidative markers. These effects can be explained by the inhibition of the iron-dependent lipid peroxidation. Additionally, DEF-induced suppression of *NCOA4* resulted in *FTH1* suppression and inhibition of lysosomal degradation of ferritin. A recent study conducted by Kim et al. (39) found comparable outcomes of protection against cisplatin-induced ferroptosis via activation of farnesoid X receptor. The authors concluded that suppression of the expression of ferroptosis-induced genes can prevent kidney injury induced by cisplatin (39,40).

These findings clarify the potential favorable role of ferroptosis suppression as a new strategy to minimize cisplatin-induced nephrotoxicity and improve the therapeutic outcome of this highly needed chemotherapeutic agent.

Although our study has introduced valuable insights into the role of ferroptosis in cisplatin-induced nephrotoxicity, the study still has limitations. Firstly, estimation of GPX4, FTH1, NCOA4, and SLC7A11 via western blot would give solid direct evidence for the presence of those specific ferroptosis key regulatory proteins. Accordingly, these specific mediators will be further explored via western blot in our ongoing research. Another limitation of this study is that *NCOA4* is crucial for degradation of ferritin through ferritinophagy, thus it would be very important to check if the autophagic process is modulated by cisplatin treatment in our future research.

### Conclusion

Overall, our study provided important new knowledge on the pathogenic mechanism responsible for iron overload-induced nephrotoxicity. We speculated that ferritin is responsible for iron release. Importantly, the activation of *NCOA4*-induced ferritinophagy could be proposed as a possible mechanism in iron overload-induced ferroptosis as well as upregulation of *IREB2*. DEF therapy may ameliorate ferritinophagy-induced iron overload and protect from ferroptosis through its iron chelation and antioxidant and anti-inflammatory properties. Our results may provide a new target for prevention against ferroptotic renal cell death in response to cisplatin.
